# Diagnostic Accuracy of Raman Spectroscopy for Oral Potentially Malignant Disorders: A Systematic Review and Meta-Analysis

**DOI:** 10.3390/diagnostics16050648

**Published:** 2026-02-24

**Authors:** Xiting Xiang, Xing Li, Zhihui Zhu, Qing Sun, Nuo Hu, Tao Zhang

**Affiliations:** 1 Division of Maxillofacial Surgery, Department of Stomatology, Peking Union Medical College Hospital, Chinese Academy of Medical Science & Peking Union Medical College, Beijing 100730, China; xiang_xiting@163.com (X.X.); lixing@student.pumc.edu.cn (X.L.); zhuzhihui@pumch.cn (Z.Z.); sunqing9701@student.pumc.edu.cn (Q.S.); hunuo111@163.com (N.H.); 2 Department of Plastic and Reconstructive Surgery, Peking Union Medical College Hospital, Chinese Academy of Medical Science & Peking Union Medical College, Beijing 100730, China

**Keywords:** Raman spectroscopy technology, oral potentially malignant disorders, oral squamous cell carcinoma, diagnostic meta-analysis, systematic review

## Abstract

**Objective**: The aim of this study was to summarize the accuracy and efficacy of Raman spectroscopy (RS) technology in identifying Oral Potentially Malignant Disorders (OPMDs). **Methods**: This systematic review was registered in PROSPERO (CRD420251124866). A literature search was conducted in databases including PubMed, Embase, and Web of Science from inception up to September 2025. The risk of bias of included studies was evaluated using the QUADAS-2 tool. Appropriate statistical techniques were applied to perform heterogeneity analysis, subgroup analyses, and calculation of pooled diagnostic efficacy indices. **Results**: A total of 12 studies involving 2810 samples were included. In the “OPMDs vs. Normal” group, RS achieved a pooled sensitivity of 0.84 (95% CI: 0.78–0.88), specificity of 0.90 (95% CI: 0.73–0.97), and AUC of 0.89 (95% CI: 0.86–0.91). In the “OPMDs vs. Oral squamous cell carcinoma (OSCC)” group, the pooled sensitivity was 0.89 (95% CI: 0.82–0.94), specificity was 0.91 (95% CI: 0.85–0.95), and AUC was 0.94 (95% CI: 0.91–0.96). Heterogeneity analysis revealed low heterogeneity in the “OPMDs vs. OSCC” group (I^2^ = 22.8%) and moderate heterogeneity in the “OPMDs vs. Normal oral mucosa” group (I^2^ = 58.9%). Subgroup analyses were performed: sample type exerted no significant impact on heterogeneity (*p* > 0.19), while Raman type (micro vs. fiber) showed potential to modulate diagnostic efficacy, micro-Raman had higher sensitivity (0.916 vs. 0.811), and fiber-Raman had better specificity (0.917 vs. 0.843). No significant publication bias was observed (Egger’s *p* > 0.3). **Conclusions**: Raman spectroscopy is an effective and reliable tool for screening and differentiating OPMDs.

## 1. Introduction

Oral squamous cell carcinoma (OSCC) accounts for approximately 90% of oral cancer, making it the thirteenth most common cancer globally [1]. Epidemiological data indicates that 389,846 new cases of oral and mouth cancer were reported in 2022 [2]. The irritation of the oral mucosa by substances such as alcohol, tobacco, betel nuts, HPV infection, and inadequate dental restorations results in an increased incidence of malignant transformation. Oral cancer usually progresses by a sequence of histological alterations, starting with normal mucosa and progressing to mild to severe atypical hyperplasia [3] and oral squamous cell carcinoma. In this carcinogenic process, Oral Potentially Malignant Disorders (OPMDs) act as the key intermediate link between normal oral mucosa and malignant tumors [4]; the most frequent types include oral leukoplakia (OLK), erythroplakia, proliferative verrucous leukoplakia (PVL), oral submucous fibrosis (OSMF), oral lichenoid diseases, graft-versus-host disease, and actinic cheilitis. These OPMDs exhibit heterogeneous malignant transformation rates, ranging from approximately 0.44% (e.g., oral lichen planus) to 43.87% (e.g., PVL) [5]. Given this variability, there is an immediate clinical necessity to explore and create innovative detection technologies that can enable early diagnosis and treatment of OPMDs, hence improving patient outcomes.

Surgical biopsy, in conjunction with histopathological diagnosis, is regarded as the gold standard [6] to evaluate suspicious oral lesions. Nevertheless, because of its invasive characteristics, this method is inappropriate for the early identification of OPMDs, especially when such lesions manifest in apparently “normal” or asymptomatic oral mucosa. There is a pressing necessity to investigate and advance novel detection technologies that enable early diagnosis and treatment of OPMDs, thus enhancing the prognosis for patients with oral cancer.

Raman spectroscopy (RS) is a non-elastic light scattering technique regarded as a potential diagnostic approach, capable of acquiring extensive molecular information from biological tissues, referred to as “molecular fingerprints.” In the carcinogenesis process within biological tissues, internal proteins, nucleic acids, lipids, carbohydrates, and other biomolecules experience alterations in conformation and amount, resulting in changes in Raman frequency shifts, peak intensities, and spectrum bands [7]. In recent years, numerous RS techniques have been extensively utilized for the differential detection of ex vivo oral cancer tissues and normal tissues [8,9,10,11]. Clinically, RS shows considerable promise in early risk stratification of suspicious oral lesions, streamlining clinical triage, and guiding precise biopsy targeting, serving as a potentially valuable adjunctive diagnostic tool to histopathological examination.

To date, a substantial body of literature has confirmed the diagnostic accuracy of Raman spectroscopy (RS) in head and neck carcinomas, with specific studies investigating its in vivo performance in oral cancer diagnosis [9,12,13,14]. For instance, Zhan (2020) [15] conducted a meta-analysis of 41 studies and reported a pooled sensitivity of 0.91 and specificity of 0.85 for in vivo oral cancer diagnosis using Raman spectroscopy (RS) [10]. Notably, this study also separately included 15 studies focusing on OPMDs, with subgroup analysis demonstrating excellent diagnostic performance of RS for OPMDs: the pooled diagnostic odds ratio (DOR) was as high as 839.25 (95% CI [274.50, 2565.92]), and the area under the summary receiver operating characteristic (SROC) curve (AUC) was 0.9932, confirming its robust potential for OPMD identification. Similarly, Shrivastava (2023) [16] performed a meta-analysis of 8 studies and reported that RS achieved a pooled sensitivity of 1.00 (95% CI: 0.72–1.00) and specificity of 0.93 (95% CI: 0.80–0.98) for detecting oral malignant and potentially malignant conditions.

However, despite these promising findings, existing meta-analyses on RS and OPMDs have not evaluated RS’s efficacy in distinguishing OPMDs from oral squamous cell carcinoma (OSCC), nor have they systematically assessed its overall diagnostic value in differentiating OPMDs, normal oral mucosa, and OSCC. Identifying the malignant transformation of OPMDs is clinically crucial for improving early cancer screening and reducing unnecessary invasive procedures, yet this remains an unmet clinical need inadequately addressed by prior research. Therefore, this meta-analysis aims to systematically evaluate the diagnostic accuracy of RS for the rapid identification of OPMDs and the differentiation of their malignant progression, clarify its efficacy in distinguishing OPMDs from normal oral mucosa and OSCC, fill the aforementioned research gap, and support the early detection of oral mucosal carcinogenesis.

## 2. Materials and Methods

This meta-analysis adhered to the Preferred Reporting Items for Systematic Reviews and Meta-Analyses (PRISMA) guidelines. A completed PRISMA 2020 checklist is provided as Supplementary Materials. The study was registered under PROSPERO number: CRD420251124866.

This systematic review and meta-analysis was guided by a clearly defined PICO (Population, Index test, Comparator, Outcomes) framework to ensure transparency, reproducibility, and methodological rigor in diagnostic accuracy assessment.

Population (P): Patients with OPMDs, OSCC, or normal oral mucosa, regardless of age, gender, OPMD subtype, or study setting.

Index test (I): Raman spectroscopy (RS), including micro-Raman and fiber-Raman systems, used for the detection and differentiation of oral mucosal lesions.

Comparator (C): Histopathological diagnosis (gold standard) for OPMDs and OSCC; normal oral mucosa as a reference group; and direct comparison between OPMDs and OSCC.

Outcomes (O): Primary outcomes: Pooled diagnostic accuracy measures including sensitivity, specificity, positive likelihood ratio (PLR), negative likelihood ratio (NLR), and area under the summary receiver operating characteristic (SROC) curve (AUC).

Secondary outcomes: Subgroup performance by Raman system type, sample type, and sources of heterogeneity.

### 2.1. Search Strategy

A systematic search of four databases—PubMed, Embase, Web of Science, and the Cochrane Library—was conducted through September 2025 to identify studies assessing the accuracy of RS in diagnosing OPMDs. Taking the PubMed database as an example, the search expression was: ((“Precancerous Conditions”[MeSH Terms] AND “Oral”[Title/Abstract]) OR “Leukoplakia, Oral”[MeSH Terms] OR “Erythroplasia”[MeSH Terms] OR “Oral Submucous Fibrosis”[MeSH Terms] OR “Lichen Planus, Oral”[MeSH Terms] OR “Oral Premalignant Lesions”[Title/Abstract] OR “Oral Potentially Malignant Disorders”[Title/Abstract] OR “Oral Dysplasia”[Title/Abstract]) AND “Spectrum Analysis, Raman”[MeSH Terms]. In addition to electronic database searches, a manual search was conducted of reference lists from previously published systematic reviews and meta-analyses on Raman spectroscopy in oral oncology (identified via initial database searches) to identify potentially eligible studies missed by electronic searches.

### 2.2. Selection Criteria

Inclusion criteria: (1) original research studies; (2) studies focusing on specimens from patients with OPMDs; (3) studies employing histopathology as the diagnostic gold standard; (4) studies utilizing RS as a diagnostic tool for screening, assessing lesion risks, delineating surgical margins, and/or guiding subsequent actions; (5) studies conducting tests on specimens from human bodies, ex vivo human tissues, or human body fluids; (6) studies providing the number of samples in each test group along with true positive (TP), false positive (FP), true negative (TN), and false negative (FN) test results.

Exclusion criteria: (1) case reports, case series, review articles, editorials, letters, comments, conference proceedings, or non-original articles; (2) studies with low sample sizes (normal/benign < 5; OPMDs/OSCC < 5); (3) studies based on animal models and cell lines; (4) studies focusing on specimens outside the oral region; (5) studies without histopathological diagnosis for the collected samples.

### 2.3. Data Extraction

Employing a uniform data extraction template, two reviewers conducted independent data retrieval from each included article and appraised its quality, resolving any disagreements through consensus. The following data were extracted: author name; publication year; patient count; sample type; spectral details; diagnostic criteria; the RS methodology of the primary investigator; the numbers of true positives (TP), true negatives (TN), false positives (FP), and false negatives (FN); and various methodological and technical specifics. The quality of each study was assessed in accordance with the Quality Assessment of Diagnostic Accuracy Studies-2 (QUADAS-2) [17].

### 2.4. Statistical Analysis

Data analysis was conducted using Stata 18.0, Meta-Disc 2.0, and Revman 5.3 software. Bivariate random-effects models were consistently applied across all diagnostic meta-analyses to synthesize the diagnostic accuracy indices. Sensitivity, specificity, and their 95% confidence intervals (CIs), along with the positive likelihood ratio (PLR), negative likelihood ratio (NLR), and diagnostic odds ratio (DOR), were calculated, and the area under the curve (AUC) was derived by plotting the SROC curve. The significance level for the meta-analysis was set at α = 0.05. The I^2^ metric was utilized to assess heterogeneity. A fixed-effects model was employed for meta-analysis when studies showed no statistical heterogeneity; otherwise, a random-effects model was utilized after investigating and excluding clear clinical heterogeneity sources. Deek’s funnel plot assessed publication bias.

## 3. Results

### 3.1. Literature Selection

**Figure 1:**
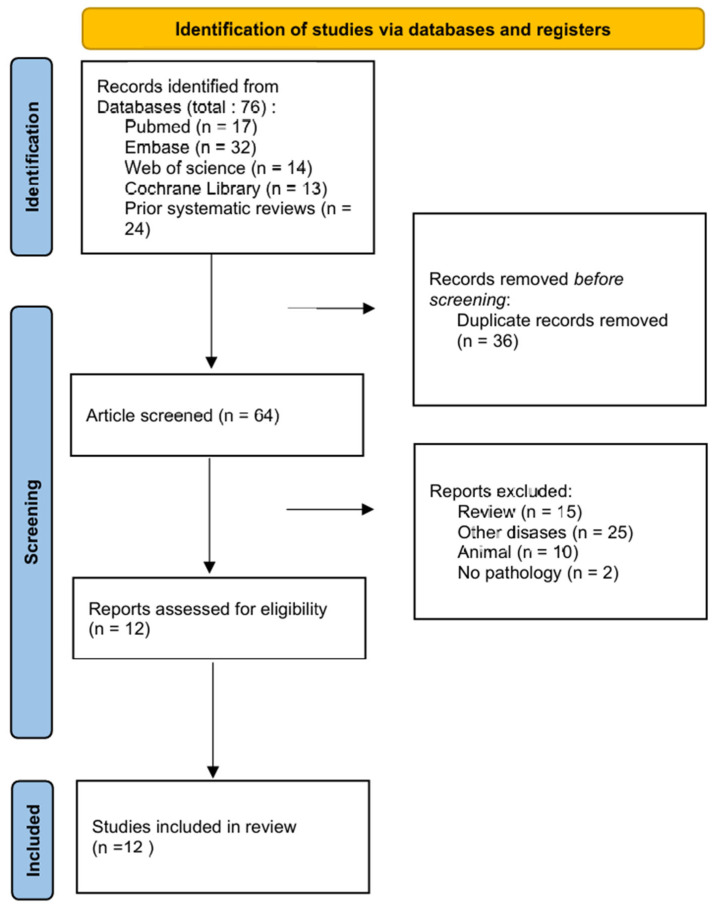
PRISMA flow chart of literature selection process.

A total of 17 articles were obtained from the PubMed database, 32 from the Embase database, 17 from the Web of Science database, and 13 from the Cochrane Library, resulting in a cumulative total of 76 papers. Following the exclusion of 12 duplicate papers, 64 papers remained. Excluding papers that lacked full text, had missing data, did not meet established criteria, were repetitive, or exhibited significant design flaws resulted in the inclusion of 12 papers [18,19,20,21,22,23,24,25,26,27,28,29] for systematic review and meta-analysis (Figure 1). A manual search of reference lists from two prior systematic reviews [15,16] and meta-analyses was performed. Zhan (2020) [15] included 16 references and Shrivastava (2023) [16] included 8 references, all of which were duplicates of studies already identified in the initial screening. After excluding non-original studies, studies without a gold standard, and studies with duplicate data, no new eligible studies were included. The article selection and systematic review process adhered to the PRISMA guidelines [17].

Table 1 summarizes the study’s characteristics of included articles, including author, publication year, sample type and corresponding sample sizes, RS parameters, and data processing methods. Among these studies, 11 provided diagnostic data for differentiating Oral Potentially Malignant Disorders (OPMDs) from oral squamous cell carcinoma (OSCC), while another 11 documented data for distinguishing OPMDs from normal samples.

### 3.2. Risk and Publication Bias Assessment

**Figure 2:**
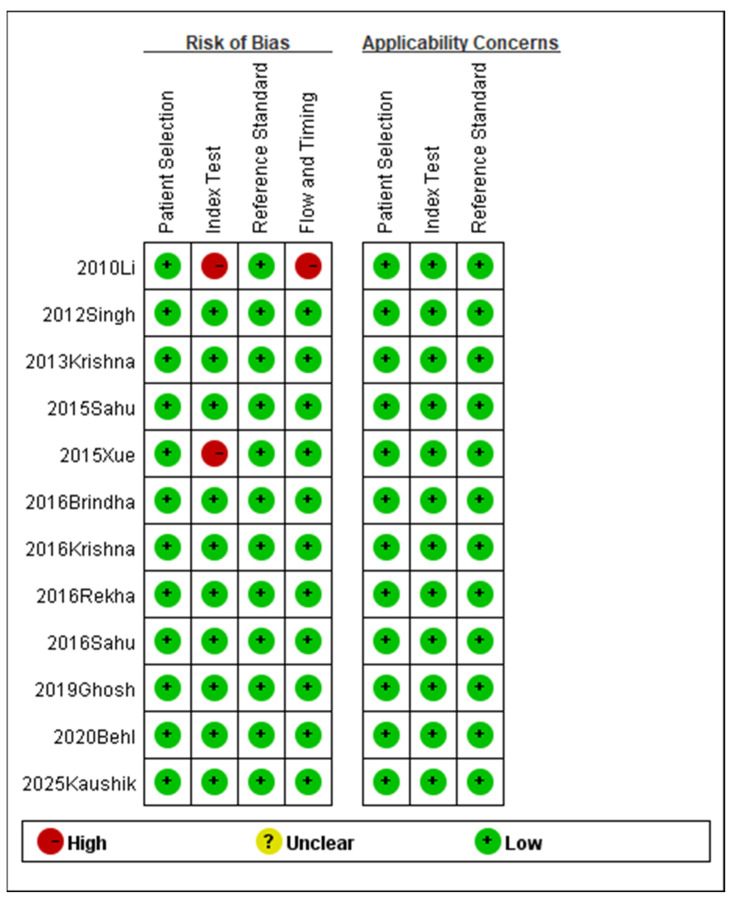
Methodological quality summary (2010 Li [18], 2012 Singh [19], 2013 Krishna [20], 2015 Sahu [21], 2015 Xue [22], 2016 Brindha [25], 2016 Krishna [23], 2016 Rekha [26], 2016 Sahu [24], 2019 Ghosh [27], 2020 Behl [28], 2025 Kaushik [29]).

The relevant data and information from the included studies were systematically organized and analyzed utilizing the QUADAS-2 tool. Figure 2 presents the evaluations of bias risk and applicability concerns across the included studies. The majority of included studies exhibited a low risk of bias across all QUADAS-2 domains, while a minority indicated a high risk of bias in certain domains.

**Figure 3:**
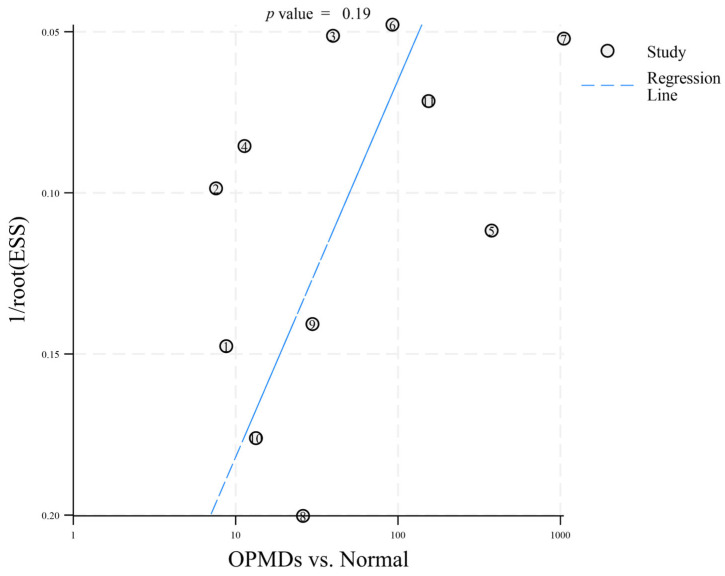
Deeks’ funnel plot asymmetry test (OPMDs vs. Normal). The numbers 1–11 correspond to references [18,19,20,21,22,23,24,25,26,27,29], respectively.

**Figure 4:**
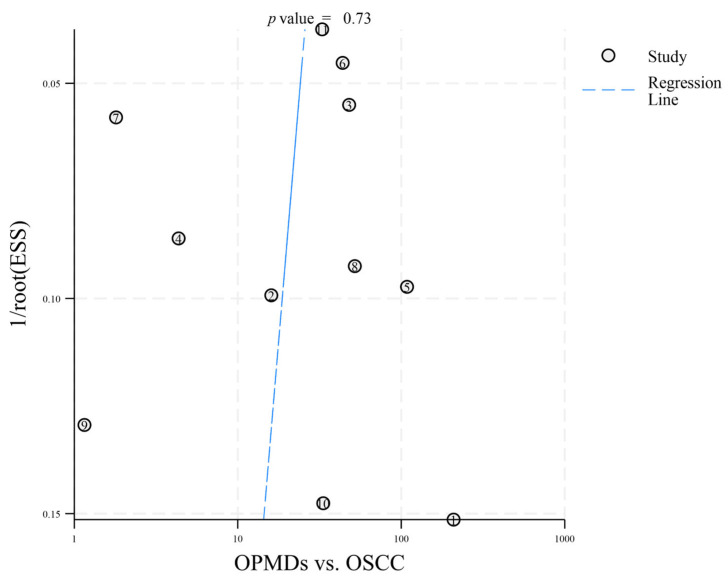
Deeks’ funnel plot asymmetry test (OPMDs vs. OSCC). The numbers 1–11 correspond to references [18,19,20,21,22,23,24,25,26,27,28], respectively.

To identify potential publication bias in the 12 included articles, distinct funnel plots were created for two comparisons: OPMDs vs. Normal (Figure 3) and OPMDs vs. OSCC (Figure 4). In the comparison of OPMDs vs. OSCC, Deeks’ funnel plot asymmetry test produced a *p*-value of 0.73. For the comparison of OPMDs vs. Normal, the *p*-value was 0.19. Both results demonstrated the absence of significant publication bias in each comparison. Egger’s Test and analogous asymmetry tests demonstrate limited statistical power with a small number of included studies. The inclusion of merely 12 studies in this research may undermine the sensitivity of the test. The slight asymmetry observed in the funnel plot suggests that the potential for publication bias cannot be entirely dismissed. The lack of gray literature, including unpublished studies and conference abstracts, may influence the results.

The findings of this meta-analysis indicate a low risk of publication bias, suggesting that the research conclusions are relatively reliable. Future research must incorporate additional high-quality studies for further verification.

### 3.3. Pooled Diagnostic Accuracy Results

#### 3.3.1. OPMDs vs. Normal

A total of 11 out of 12 included studies were eligible for meta-analysis regarding the differentiation of OPMDs from normal oral mucosa. Complete data extraction was achieved for true positive (TP), false positive (FP), true negative (TN), and false negative (FN) across all eligible studies.

**Figure 5:**
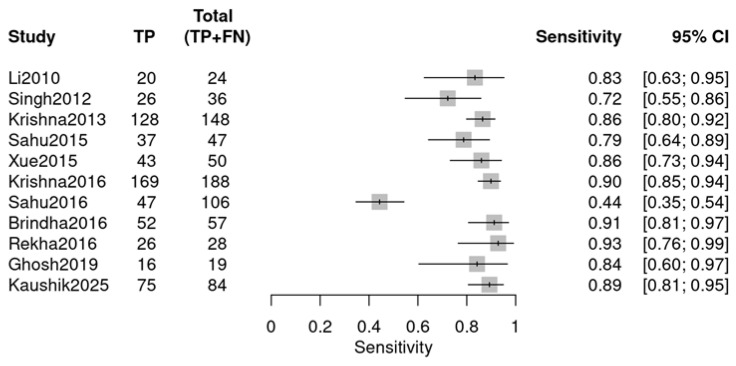
Sensitivity forest plot (OPMDs vs. Normal) (Li 2010 [18], Singh 2012 [19], Krishna 2013 [20], Sahu 2015 [21], Xue 2015 [22], Krishna 2016 [23], Sahu 2016 [24], Brindha 2016 [25], Rekha 2016 [26], Ghosh 2019 [27], Kaushik 2025 [29]).

**Figure 6:**
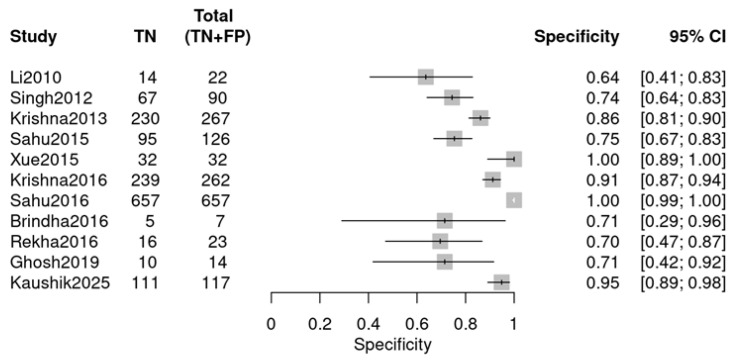
Specificity forest plot (OPMDs vs. Normal) (Li 2010 [18], Singh 2012 [19], Krishna 2013 [20], Sahu 2015 [21], Xue 2015 [22], Krishna 2016 [23], Sahu 2016 [24], Brindha 2016 [25], Rekha 2016 [26], Ghosh 2019 [27], and Kaushik 2025 [29]).

Individual studies exhibited marked variability in diagnostic performance, as visualized by two forest plots. For sensitivity (Figure 5), values ranged from 0.44 (Sahu 2016 [24]) to 0.93 (Rekha 2016 [26]). For specificity (Figure 6), the range was 0.64 (Li 2010 [18]) to 1.00 (Xue 2015 [22], Sahu 2016 [24]). The observed variability suggests potential heterogeneity, likely due to variations in study design, sample type, or RS parameters.

Using a bivariate random-effects meta-analysis model, the pooled diagnostic efficacy indicators for OPMDs vs. Normal, including sensitivity, specificity, diagnostic odds ratio (DOR), positive likelihood ratio (PLR), and negative likelihood ratio (NLR), are summarized in Table 2. These indicators illustrate the strong discriminative performance of RS. The pooled sensitivity of 0.843 reduces missed screenings for OPMDs, confirming its effectiveness as an initial detection tool. Additionally, the pooled specificity of 0.900 (FPR = 0.100) decreases the likelihood of unnecessary invasive procedures for individuals with normal oral mucosa. Additionally, high pooled DOR (48.359) attests to strong overall diagnostic efficacy, while elevated pooled PLR (8.414) and low pooled NLR (0.174) strengthen confidence in positive result validity and negative-case exclusion reliability.

**Figure 7:**
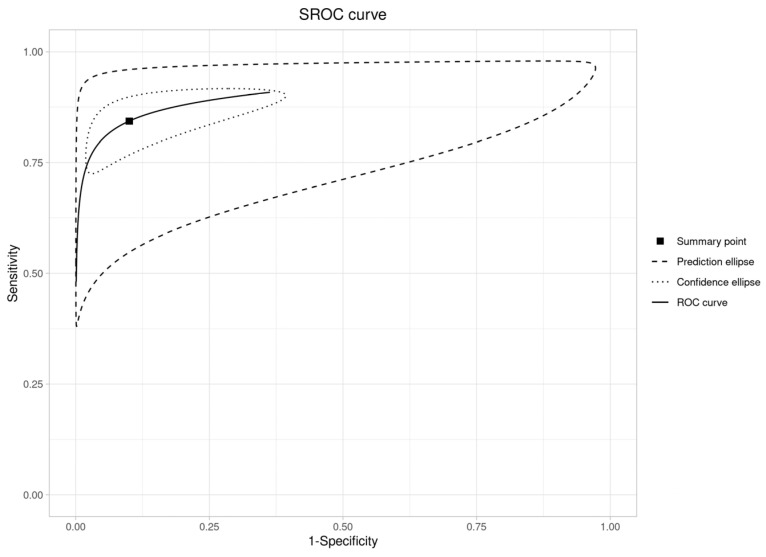
SROC curve plot (OPMDs vs. Normal).

An SROC curve was created to assess diagnostic abilities (Figure 7), resulting in an area under the curve of 0.91 (95% CI: 0.88–0.93). This is “excellent” (AUC > 0.90), validating RS’s ability to distinguish OPMDs from normal oral mucosa.

#### 3.3.2. OPMDs vs. OSCC

**Figure 8:**
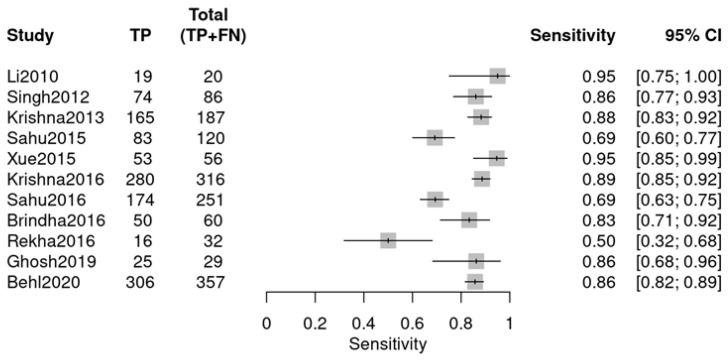
Sensitivity forest plot (OPMDs vs. OSCC) (Li 2010 [18], Singh 2012 [19], Krishna 2013 [20], Sahu 2015 [21], Xue 2015 [22], Krishna 2016 [23], Sahu 2016 [24], Brindha 2016 [25], Rekha 2016 [26], Ghosh 2019 [27], Behl 2020 [28]).

**Figure 9:**
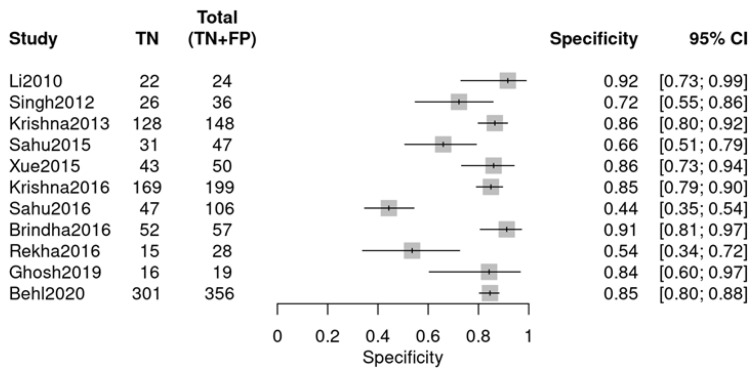
Specificity forest plot (OPMDs vs. OSCC) (Li 2010 [18], Singh 2012 [19], Krishna 2013 [20], Sahu 2015 [21], Xue 2015 [22], Krishna 2016 [23], Sahu 2016 [24], Brindha 2016 [25], Rekha 2016 [26], Ghosh 2019 [27], Behl 2020 [28]).

This group comprised 11 studies from a total of 12, with one study excluded. Data extraction adhered to the same methodology and encompassed similar diagnostic data as the previous group (Figure 8 and Figure 9). The individual studies demonstrated variability in diagnostic performance: sensitivity varied from 0.50 (Rekha 2016 [26]) to 0.95 (Li 2010 [18]), and specificity ranged from 0.44 (Sahu 2016 [24]) to 0.94 (Brindha 2016 [25]), as illustrated in the forest plots.

The combined diagnostic effectiveness demonstrates significant clinical value (Table 3). A pooled sensitivity of 0.838 facilitates the accurate identification of approximately 84% of cases, thereby minimizing the risk of missed diagnoses and endorsing its application in screening processes. The pooled specificity of 0.791 indicates that approximately 79% of non-cases are excluded. A DOR of 19.61, significantly greater than 1, indicates strong overall discriminatory ability. A PLR of 4.018 indicates that a positive result is approximately four times more likely in cases, thereby enhancing confidence in positive findings. An NLR of 0.205 suggests that a negative result is approximately five times more probable in non-cases, thereby rendering negative results highly dependable for the exclusion of disease. In summary, RS exhibits robust comprehensive performance, notably excelling in sensitivity and overall differentiation.

**Figure 10:**
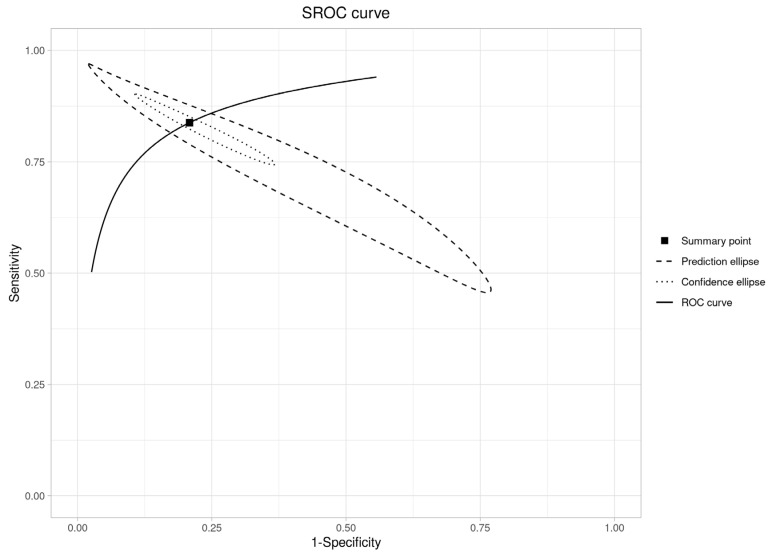
SROC curve plot (OPMDs vs. OSCC).

The SROC curve resides in the upper-left region (Figure 10), consistent with an AUC of 0.89 (95% CI: 0.86–0.91), a value that falls into the “good” category (typically defined as AUC 0.8–0.9) and confirms RS’s robust ability to differentiate OPMDs from OSCC.

#### 3.3.3. Cross-Group Comparison

To clarify RS’s performance across scenarios, we compared pooled diagnostic efficacy between the “OPMDs vs. Normal” and “OPMDs vs. OSCC” groups. The “OPMDs vs. OSCC” group exhibited higher sensitivity (0.89 vs. 0.84), specificity (0.91 vs. 0.90), DOR (85.2 vs. 48.4), and AUC (0.94 vs. 0.89), indicating superior efficacy in differentiating OPMDs from OSCC than from normal mucosa.

**Figure 11:**
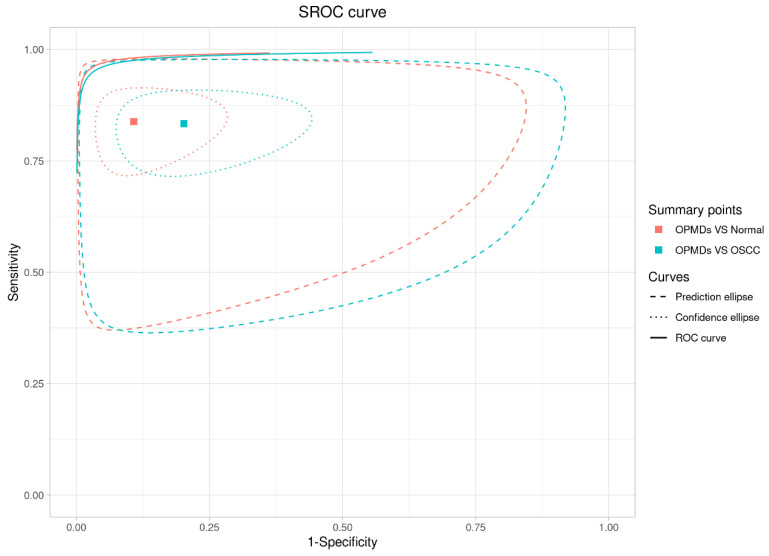
Combined analysis of the SROC curve.

A combined analysis of the SROC curve (Figure 11) and meta-regression results reveals important details: although the “OPMDs vs. Normal” curve (red) is positioned nearer to the upper-left region, meta-regression indicated no statistically significant differences in relative sensitivity (*p* = 0.918), relative specificity (*p* = 0.197), or global test comparison (*p* = 0.425) between the two groups. Despite this statistical parity, RS retains distinct value in the “screening to differentiation” workflow: it serves as a dependable preliminary screening tool for OPMDs compared to normal mucosa, utilizing high sensitivity (0.84) and AUC (0.89) to reduce missed diagnoses. In differentiating OPMDs from OSCC, RS also exhibits favorable specificity (0.91), DOR (85.2), and AUC (0.94), facilitating accurate treatment decisions (e.g., conservative management for OPMDs versus radical therapy for OSCC). RS facilitates thorough management of oral lesions, encompassing detection and accurate classification, and demonstrates consistent performance in various clinical contexts.

#### 3.3.4. Heterogeneity and Subgroups Analysis

The heterogeneity analysis (Table 4) revealed that the bivariate I^2^ statistic was 22.8% for the OPMDs vs. OSCC group, reflecting low heterogeneity, while it reached 58.9% for the OPMDs vs. Normal group, suggesting moderate heterogeneity. In terms of variance components, the variance of logit-transformed sensitivity (Var logit(sen) = 0.349) and specificity (Var logit(spe) = 0.686) in the OPMDs vs. OSCC group were both lower than those in the OPMDs vs. Normal group (0.494 and 3.529, respectively), indicating less between-study variation in the former. The multiplicative odds ratios (MORs) for sensitivity (1.757) and specificity (2.203) in the OPMDs vs. OSCC group were also significantly lower than those in the OPMDs vs. Normal group (1.955 and 6.001, respectively), further confirming weaker heterogeneity in the former. Additionally, the area of the 95% prediction ellipse was much smaller in the OPMDs vs. OSCC group (0.068) than in the OPMDs vs. Normal group (0.26), intuitively reflecting better consistency of results. In conclusion, RS exhibits lower between-study heterogeneity and stronger result stability in the “OPMDs vs. OSCC” scenario, while there is moderate heterogeneity in the “OPMDs vs. Normal” scenario, which requires further exploration of sources through subgroup analysis.

To identify the potential drivers of moderate heterogeneity in the “OPMDs vs. Normal” group, we conducted subgroup analyses based on type of sample (Table 5) and type of RS (Table 6), respectively, with results as follows:

The tissue subgroup demonstrated superior performance in specificity and DOR. Subsequent meta-regression analysis indicated that the *p*-value for relative sensitivity level (tissue vs. other) was 0.194, that for relative specificity level was 0.197, and that for global test comparison was 0.349. All *p*-values were >0.05, indicating that “type of sample” had no statistically significant impact on the heterogeneity of this group.

The micro subgroup performed better in sensitivity, while the fiber subgroup was superior in specificity. Meta-regression analysis showed that the *p*-value for relative sensitivity level (micro vs. fiber) was 0.086 (approaching statistical significance), that for relative specificity level was 0.586, and that for global test comparison was 0.186. This suggests that the type of RS may potentially affect sensitivity.

## 4. Discussion

This study assessed the diagnostic efficacy of RS in differentiating OPMDs from normal oral mucosa and OSCC, emphasizing the resolution of clinical unmet needs and methodological deficiencies in previous research.

The diagnostic efficacy of RS aligns with the clinical workflow in oral oncology, and its potential clinical scenarios are clearly defined herein to enhance translational relevance. Specifically, RS is well-suited for three core clinical settings: First, population-based preliminary screening in primary care or community health centers, where its high sensitivity (0.84) can effectively identify suspicious lesions in asymptomatic individuals or those with a high risk factor, addressing the issue of overlooked early lesions when pathological biopsy is not readily accessible. Second, it serves as a pre-biopsy adjunctive tool during specialist assessment in stomatology departments, as its favorable specificity (0.90) helps triage suspicious lesions, minimizing unnecessary invasive biopsies and alleviating patient anxiety. Third, it holds promise for long-term follow-up of patients with OPMDs, enabling non-invasive dynamic monitoring of lesion progression and aiding in timely identification of malignant transformation. The enhanced efficacy for differentiating OPMDs from OSCC aids in addressing the significant challenges of over-treatment and under-treatment in contemporary clinical practice. The lack of statistically significant efficacy differences between the two scenarios (global *p* = 0.425) further underscores RS’s adaptability across these clinical stages.

Notably, our pooled diagnostic accuracy (sensitivity = 0.84, specificity = 0.90) for differentiating OPMDs from normal mucosa and OSCC is consistent with the findings of Zhan (2020) [15], who reported a pooled sensitivity of 0.96 and specificity of 0.90 for RS in diagnosing oral precancerous lesions [10]. This alignment validates RS’s reproducible diagnostic value in oral mucosal lesions, while our study extends their work by specifically clarifying RS’s efficacy in distinguishing OPMDs from OSCC. This critical aspect was not addressed in their meta-analysis and fills a key research gap in the field.

Heterogeneity analysis identifies key patterns: the low heterogeneity in differentiating OPMDs from OSCC (I^2^ = 22.8%) is due to OSCC’s unique pathology causing consistent spectral changes, while moderate heterogeneity in distinguishing OPMDs from normal tissue (I^2^ = 58.9%) is linked to normal mucosal variations. Subgroup analyses indicate that sample type does not affect heterogeneity (*p* > 0.19), supporting RS’s non-invasive use. Raman type is a modifiable factor; micro-Raman is more sensitive (0.916 vs. 0.811) for early lesions, and fiber-Raman is more specific (0.917 vs. 0.843) for bedside screening.

Important limitations of this investigation should be acknowledged. We fully recognize these constraints and their potential impact on the precision, robustness, and interpretability of the pooled findings. First, stratified analysis according to OPMD subtypes could not be performed, largely due to incomplete reporting of subtype classifications and the general absence of prospective, subtype-stratified research designs in the majority of included primary studies. Given the divergent malignant potential across distinct OPMDs entities, this may reduce the specificity and granularity of RS diagnostic efficacy estimates. Second, inconsistent and incomplete reporting of key sample and clinical characteristics—including histopathological differentiation of oral cancer, patient lifestyle habits, and detailed sample source information—limited further exploratory subgroup analyses and may affect the external generalizability of the synthesized results. Third, the micro-Raman subgroup comprised only three included studies, which reduces statistical precision and restricts the stability and generalizability of corresponding subgroup estimates.

Despite a comprehensive search and screening strategy implemented across multiple databases and relevant prior reviews, the overall number of high-quality diagnostic accuracy studies eligible for inclusion remains relatively modest. This largely reflects the current breadth and maturity of peer-reviewed evidence focused on RS for OPMDs diagnosis. To strengthen future evidence in this field, subsequent research is encouraged to implement standardized OPMD subtype categorization, adopt prospectively planned subtype-stratified diagnostic evaluations, improve uniformity in reporting sample and patient-level characteristics, expand sample sizes in underrepresented technical subgroups, and perform head-to-head comparative studies between RS and established non-invasive diagnostic modalities. These steps may better define the clinical performance and implementation potential of RS for OPMD detection and risk stratification.

## 5. Conclusions

This study validates that RS technology shows promising diagnostic performance for OPMDs, offering non-invasive, real-time, and rapid identification capabilities that support a range of adjunctive clinical applications. Nonetheless, important limitations persist that require further refinement. In conclusion, RS holds potential as a complementary screening, triage, or biopsy-guidance tool for OPMDs, but should not be regarded as a replacement for histopathological diagnosis (the current gold standard). Ongoing research and development are needed to advance its clinical translation and standardization.

## Figures and Tables

**Table 1:** Study characteristics.

Study ID	Author	Year	Type of Sample	Sample Size			Lesion Type	Type of Raman	Excitation Wavelength	Spectral Range	Integration Time	Data Processing Method		Pre Mal and OSCC				Pre Mal and Normal			
				Nomal	OPMDs	OSCC						Algorithm	Model Validation Method	TP	FP	TN	FN	TP	FP	TN	FN
1	Li [18]	2010	Tissue samples	28	32	58	OLK	fiber	1064 nm	100–3800 cm^−1^	-	SVM	LOOCV and blind calibration	19	2	22	1	20	8	14	4
2	Singh [19]	2012	Tissue samples	50	24	50	-	fiber	785 nm	1200–1800 cm^−1^	3 s	PCA-LDA	LOOCV and independent test	74	10	26	12	26	23	67	10
3	Krishna [20]	2013	Tissue samples	26	44	69	OLK, OSMF	fiber	785 nm	900–1750 cm^−1^	5 s	Sparse Multinomial Logistic Regression, SMLR	LOOCV (Leave-One-Subject-Out Cross-Validation)	165	20	128	22	128	37	230	20
4	Sahu [21]	2015	Serum	126	47	120	OLK, OSMF	fiber	785 nm	700–1800 cm^−1^	15 s	PC-LDA	LOOCV	83	16	31	37	37	31	95	10
5	Xue [22]	2015	Tissue samples	32	50	56	Severe Dysplasia	fiber	785 nm	600–1800 cm^−1^	40 s	PCA-DFA	cross-validation	53	7	43	3	43	0	32	7
6	Krishna [23]	2016	Tissue samples	28	58	113	OLK, OSMF	fiber	785 nm	950–1750 cm^−1^	5 s	the MRDF-SMLR diagnostic algorithm	LOOCV	280	30	169	36	169	23	239	19
7	Sahu [24]	2016	Tissue samples	72	40	85	OLK, OSMF	fiber	785 nm	1200–1800 cm^−1^	3 s	PC-LDA	LOOCV	174	59	47	77	47	0	657	59
8	Brindha [25]	2016	Urine	80	57	60	-	micro	785 nm	2600–3500 cm^−1^	90 s	PC-LDA	-	50	5	52	10	52	2	5	5
9	Rekha [26]	2016	Saliva	23	28	32	OSMF	micro	784.15 nm	200–1800 cm^−1^	120 s	PCA-LDA	LOOCV	16	13	15	16	26	7	16	2
10	Ghosh [27]	2018	Oral exfoliated cells	11	13	10	OLK	fiber	785 nm	400–1800 cm^−1^	50 s	PCA–LDA	k-fold	25	3	16	4	16	4	10	3
11	Behl [28]	2020	Oral brush biopsy cytological samples	20	20	-	OLK, erythroplakia	micro	532 nm	400–1800 cm^−1^	30 s	PLS-DA	LOOCV	-				306	55	301	51
12	Kaushik [29]	2025	Saliva	32	28	39	-	micro	785 nm	400–2200 cm^−1^	20–30 s	PLS-DA	-	75	6	111	9	-			

**Table 2:** Pooled diagnostic efficacy indicators (OPMDs vs. Normal).

	Estimate	95% LCI	95% UCI
Sensitivity	0.843	0.769	0.897
Specificity	0.9	0.733	0.967
DOR	48.359	18.355	127.409
PLR	8.414	3.033	23.341
NLR	0.174	0.123	0.246
FPR	0.1	0.033	0.267

**Table 3:** Pooled diagnostic efficacy indicators (OPMDs vs. OSCC).

	Estimate	95% LCI	95% UCI
Sensitivity	0.838	0.778	0.884
Specificity	0.791	0.691	0.866
DOR	19.61	8.032	47.873
PLR	4.018	2.494	6.472
NLR	0.205	0.134	0.314
FPR	0.209	0.134	0.309

**Table 4:** Heterogeneity analysis.

	OPMDs vs. OSCC	OPMDs vs. Normal
Var logit(sen)	0.349	0.494
Var logit(spe)	0.686	3.529
MOR sensitivity	1.757	1.955
MOR specificity	2.203	6.001
Bivariate I^2^	0.228	0.589
Area 95% Prediction Ellipse	0.068	0.26

**Table 5:** Subgroup analysis by type of sample.

Parameter	Estimate Other	95% LCI Other	95% UCI Other	Estimate Tissue	95% LCI Tissue	95% UCI Tissue
Sensitivity	0.884	0.79	0.94	0.804	0.695	0.88
Specificity	0.801	0.45	0.952	0.945	0.79	0.988
DOR	30.798	7.836	121.055	70.957	19.304	260.824
PLR	4.444	1.291	15.297	14.721	3.687	58.783
NLR	0.144	0.084	0.247	0.207	0.135	0.319
FPR	0.199	0.048	0.55	0.055	0.012	0.21

**Table 6:** Subgroup analysis by type of RS.

Parameter	Estimate Fiber	95% LCI Fiber	95% UCI Fiber	Estimate Micro	95% LCI Micro	95% UCI Micro
Sensitivity	0.811	0.721	0.876	0.916	0.816	0.964
Specificity	0.917	0.737	0.977	0.843	0.375	0.98
DOR	46.994	15.2	145.291	58.584	9.251	371.016
PLR	9.709	2.941	32.057	5.834	0.957	35.571
NLR	0.207	0.145	0.294	0.1	0.05	0.199
FPR	0.083	0.023	0.263	0.157	0.02	0.625

## Data Availability

This systematic review and meta-analysis is based on secondary analysis of publicly available data from previously published studies. All included primary studies are listed in the reference section, and their original data can be obtained via the journal websites or by requesting from the corresponding authors of those studies. The literature search strategy (including databases, keywords, and retrieval timeframes) and PRISMA flow diagram are presented in the main text. Additionally, the aggregated data used for meta-analysis (e.g., sensitivity, specificity, pooled effect sizes) are available from the corresponding author upon reasonable request for academic purposes.

## Supplementary Materials

The following supporting information can be downloaded at: https://www.mdpi.com/article/10.3390/diagnostics16050648/s1, File S1: PRISMA 2020 Main Checklist.

## Abbreviations

The following abbreviations are used in this manuscript:

RS	Raman Spectroscopy
OPMDs	Oral Potentially Malignant Disorders
OSCC	Oral Squamous Cell Carcinoma
PVL	Proliferative Verrucous Leukoplakia
OSMF	Oral Submucous Fibrosis
PRISMA	Preferred Reporting Items for Systematic Reviews and Meta-Analyses
TP	True Positive
FP	False Positive
TN	True Negative
FN	False Negative
CI	Confidence Interval
PLR	Positive Likelihood Ratio
NLR	Negative likelihood Ratio
DOR	Diagnostic Odds Ratio
AUC	Area Under the Curve
MOR	Multiplicative Odds Ratio
Var logit(sen)	Variance of logit-transformed sensitivity
Var logit(spe)	Variance of logit-transformed specificity
